# Cause-of-death ascertainment for deaths that occur outside hospitals in Thailand: application of verbal autopsy methods

**DOI:** 10.1186/1478-7954-8-13

**Published:** 2010-05-18

**Authors:** Warangkana Polprasert, Chalapati Rao, Timothy Adair, Junya Pattaraarchachai, Yawarat Porapakkham, Alan D Lopez

**Affiliations:** 1 School of Health Sciences, Thummathirat Open University, Bangkok, Thailand; 2 School of Population Health, University of Queensland, Brisbane, Australia; 3 Department of Community Medicine, Thammasat University, Pathumthani, Thailand; 4 Ministry of Public Health, Bangkok, Thailand; 5 Health Information Systems Knowledge Hub, University of Queensland, Brisbane, Australia

## Abstract

**Background:**

Ascertainment of cause for deaths that occur in the absence of medical attention is a significant problem in many countries, including Thailand, where more than 50% of such deaths are registered with ill-defined causes. Routine implementation of standardized, rigorous verbal autopsy methods is a potential solution. This paper reports findings from field research conducted to develop, test, and validate the use of verbal autopsy (VA) methods in Thailand.

**Methods:**

International verbal autopsy methods were first adapted to the Thai context and then implemented to ascertain causes of death for a nationally representative sample of 11,984 deaths that occurred in Thailand in 2005. Causes of death were derived from completed VA questionnaires by physicians trained in ICD-based cause-of-death certification. VA diagnoses were validated in the sample of hospital deaths for which reference diagnoses were available from medical record review. Validated study findings were used to adjust VA-based causes of death derived for deaths in the study sample that had occurred outside hospitals. Results were used to estimate cause-specific mortality patterns for deaths outside hospitals in Thailand in 2005.

**Results:**

VA-based causes of death were derived for 6,328 out of 7,340 deaths in the study sample that had occurred outside hospitals, constituting the verification arm of the study. The use of VA resulted in large-scale reassignment of deaths from ill-defined categories to specific causes of death. The validation study identified that VA tends to overdiagnose important causes such as diabetes, liver cancer, and tuberculosis, while undercounting deaths from HIV/AIDS, liver diseases, genitourinary (essential renal), and digestive system disorders.

**Conclusions:**

The use of standard VA methods adapted to Thailand enabled a plausible assessment of cause-specific mortality patterns and a substantial reduction of ill-defined diagnoses. Validation studies enhance the utility of findings from the application of verbal autopsy. Regular implementation of VA in Thailand could accelerate development of the quality and utility of vital registration data for deaths outside hospitals.

## Introduction

Death registration systems in Thailand have improved greatly over the past two decades. In 2005, the national death registration system recorded just more than 395,000 deaths, about 100,000 more than a decade earlier [1]. However, since the majority of deaths (about 65%) in Thailand occur outside hospitals and in the absence of medical attention, the reliability of causes of death at registration remains uncertain. In most cases, causes of these deaths are recorded by nonmedical civil registrars based on lay reports from relatives, occasionally informed by medical opinion obtained during the illness leading to death [2]. As a result, a very substantial fraction of deaths occurring at home are registered with ill-defined causes of death, limiting the utility of registration data for epidemiological research and health policy [3].

An investigation of registered causes of death in Thailand during 1998-1999 confirmed the potential to obtain more accurate information on causes of death through verbal autopsy (VA) methods, a process combining household inquiry with a review of medical records, where available [4,5]. Building on this previous research, we designed and implemented a nationally representative study utilizing VA methods adapted to the Thai context to ascertain the likely causes of deaths that had occurred outside hospitals during 2005. This article reports on the development of these VA methods for application in Thailand, our principal findings from implementing them in field studies, and their implications for routine monitoring of causes of death in the country.

VA methods include the use of detailed questionnaires administered by local health personnel to collect information from relatives of the deceased on symptoms and events during the illness leading to death. Completed questionnaires are subsequently reviewed by a physician, who derives the probable cause of death from the information recorded during the interview [6]. Although there are several potential sources of bias in verbal autopsies, these methods are increasingly being used to derive cause-specific mortality estimates in populations that do not have complete medical certification of cause of death [7-16]. In view of the potential for bias, VA methods in any population should first be validated to ascertain the performance characteristics of the instrument, in terms of any systematic over- or underestimation of mortality from particular causes. VA methods have been extensively validated for causes of childhood deaths [17-21]. However, validation of VA methods for adult deaths poses several challenges, particularly in terms of obtaining reference diagnoses, as well as in multiple causation and presentation of symptoms, especially for chronic diseases [22-27].

In this paper, we report on findings from the application of VA methods to ascertain causes of death in Thailand. We conducted two studies:

1) field implementation of VA methods to verify registered causes of deaths outside hospitals in the study sample (the *verification *study).

2) concurrent implementation of the same VA methods (to validate their performance characteristics) on deaths in the study sample that had occurred in hospitals, for which reference diagnoses were available from medical record review (the *validation *study).

We also report on the application of the validation study results to adjust the VA- based findings from the verification study, yielding adjusted estimates of cause-specific mortality for deaths outside hospitals in Thailand. Apart from generating these mortality estimates for health status assessment and planning in Thailand, this study has developed the foundation for strengthening routine cause-of-death ascertainment in Thailand through the capacity that has been built to implement the methods. Wider implementation of these methods over the next decade, with continuous refinement based on field experiences, could substantially improve the utility of registration data for public health policy and research in Thailand.

## Methods

### Study objectives and design

A cross-sectional study was designed to allow VA methods to be applied to ascertain causes of death in a nationally representative sample of deaths that occurred in Thailand during 2005. The study formed part of a broader research project to estimate cause-specific mortality in Thailand, as described elsewhere [28]. The objectives of this component of the overall project were to:

1. adapt recent international VA standards for implementation in Thailand [29].

2. implement the locally adapted VA methods in the *verification *and *validation *arms of the study.

3. utilize these findings to estimate cause-specific mortality fractions for deaths that occur outside hospitals in Thailand.

### Local adaptation of VA methods

VA methods have been applied before in Thailand. Perhaps the most comprehensive application was carried out in 1997-1999 using a questionnaire that consisted of: a checklist of 10 key symptoms; a checklist of common diseases among children as well as maternal conditions and external causes; and space for free-text recording of the illness preceding death as described by relatives of the deceased [4]. Wherever available, medical records pertaining to the deceased were accessed from district and/or provincial hospitals. Physician reviewers ascertained the most likely cause of death from all evidence available for each death. Causes of death from the study were subsequently used to estimate cause-specific mortality in Thailand [30]. Other smaller studies using personal digital assistant (PDA) devices have been carried out in the Kanchanaburi Demographic Surveillance site in central Thailand [31]. However, upon validation against deaths with reference diagnoses, the accuracy of this tool was disappointing due to the occurrence of false positive or negative responses that did not fit the programmed algorithms.

Building upon these experiences, we first reviewed current VA questionnaires in use in different international statistics and research programs [29,32,33]. Initially, Thai-translated versions of the draft WHO VA questionnaires (for three age groups: <28 days; 29 days - 5 years; and 5 years and above) were pilot-tested in a sample of about 400 deaths in Ayuthaya province in central Thailand. Feedback from the pilot study led to modification of the instrument design and development of Thai versions of VA questionnaires for deaths within two specific age groups: less than 1 year, and 1 year and above. The broad structure and content of the questionnaires are summarized in Table 1.

**Table 1:** Structure of Thai verbal autopsy questionnaires

Deaths under one year of age	Items	Deaths aged one year and over	Items
General information	15	General information	15
			
Basic information on mother of deceased	6	Basic information on the deceased	10
			
Basic information on the deceased	12	Accident and injuries	3
			
Accident and injuries	2	Check list of symptoms, including 11 items for maternal deaths	61
			
Check list of symptoms on pregnancy and delivery, and on child health	33	History of chronic conditions and behavioural risk factors of the deceased	3
			
Description of illness and death as told by respondents	2	Description of illness and death as told by respondents	2
			
Health records	9	Health records	9
			
Cause of death from death certification or other documents	7	Cause of death from death certification and other documents	7
			
Total items	86	Total items	110

### Sampling plan

The detailed sampling plan for the project has been described elsewhere [29]. The estimated total sample size for the study was about 10,000 deaths. A multistage stratified cluster sampling approach was employed to develop a nationally representative sample of deaths from two provinces in each of four regions of Thailand (Northeast, North, Central, and South), as well as in Bangkok. The sample was distributed according to probability proportionate to size (PPS), i.e., number of registered deaths in 2005 across three major strata: region, province, and district. Based on findings from the pilot study and previous research [4], initial PPS regional samples were inflated by 15% for each of the four regions and by more than 50% in Bangkok to account for expected losses to follow up. Within each major region, subunits were divided into two strata at the 50^th ^percentile of numbers of registered deaths, with random selection of subunits from each stratum to fulfill the allocated PPS study sample. This form of stratification was implemented to ensure representation of predominantly urban and rural communities and to account for differential access to health facilities. At the district level, the principle of random selection of deaths without replacement was applied to select the allocated PPS sample of deaths. A final total of 11,984 deaths constituted the study sample, distributed across 28 districts in nine provinces from the four regions of Thailand and the three major subdistricts of Bangkok.

### Field implementation

For each death selected into the study, the field protocols were as follows:

1. initial contact with household to obtain consent to participate in the study, to access medical records if the death had occurred in a hospital, and to arrange appointment for VA interview.

2. subsequent household visit to conduct VA interview.

3. review of completed questionnaire by a trained physician, leading to certification of cause of death using the international form of medical certificate of cause of death.

4. assignment of ICD codes to listed causes of death by trained coders.

5. final review by certifying physician to select the underlying cause of death and associated ICD code.

6. systematic assessment by the central study team of the accuracy of selection and coding of underlying causes of death, leading to revised cause-of-death choices in some cases.

In addition, for those deaths that had occurred in hospitals, an independent ascertainment of cause of death from medical records review was undertaken, as described elsewhere [34].

Data collection was conducted between June 2006 and July 2007, resulting in VA recall periods ranging from six to 18 months. Field activities were supported by specific training programs conducted in each province for VA interviewers, physicians, and medical coders. Paramedical staff members from district health offices and health centers were selected as field interviewers, according to specified criteria regarding qualifications and experience, with on-site supervision and quality assurance by experienced provincial staff.

Death certification from VA questionnaires and ICD coding were conducted by trained teams in Provincial Chief Medical Offices (PCMO). Family-medicine general practitioners were chosen to certify deaths from VA. Physician certification training programs were based on the principles of multiple causes of death using the standard international medical certificate of cause of death [35], which is consistently used throughout Thailand for certifying deaths in hospitals. The training programs included exercises to measure reliability of cause-of-death ascertainment using standard clinical case scenarios. These confirmed a high degree of concurrence in certification practices among participants. Each VA questionnaire was reviewed by a single physician, and if necessary, a second opinion was sought (in about 15% of cases), resulting in either a consensus diagnosis or, in the case of disagreement, the assignment of an ill-defined cause. During physician reviewer training programs, each participant was required to practice cause-of-death certification and ICD coding using 12 sample VA questionnaires, and retraining was conducted until satisfactory performance was achieved by all study reviewers. The criterion for satisfactory performance was that the ICD code for the underlying cause determined by the physician reviewer should match the code for the reference underlying cause for that case, at the ICD Mortality Tabulation List 1 level of aggregation of ICD codes [36].

Adequate quality control measures were adopted throughout data collection and processing, in the form of field supervision of VA interviews, manual verification of completeness of questionnaires and consistency in responses, and review of death certificates and ICD coding by a central team of experts from the Ministry of Public Health. Where necessary, adjudication by the central team was used to overrule decisions on selection of underlying causes at the provincial level.

In case the death had occurred in a hospital, a detailed review of the medical record was conducted [34]. The quality of clinical evidence supporting the medical records diagnoses was categorized as confirmatory or weak, based on the information available from the medical records. Review of medical records and VA for the same death was conducted strictly independently. All data from VA-based death certificates, the medical record review diagnoses, and essential variables from registration data, including the ICD code for the underlying cause of death, were entered into a statistical database for analysis.

### Statistical analyses

Underlying causes of death from each of the three sources (registration, VA, and medical record review) were aggregated to the WHO Mortality Tabulation List 1 [36]. Given the large proportion of deaths classified to ill-defined causes in the registration data, the allocation of these deaths to specific causes in the study sample was of primary interest. Further analyses were also conducted to assess misallocation across specified causes. The overall implications of the findings from VA ascertainment of causes of deaths outside hospitals in Thailand were assessed through an analysis of net changes to cause-specific mortality fractions for leading causes.

The validity of the VA methods used in this study was analysed in terms of the sensitivity of VA diagnoses as compared to medical records diagnoses for each of 2,558 deaths for which medical records were available. Performance characteristics of VA in terms of over- or underdiagnoses of specific causes of death were assessed through analyses of net differences in cause-specific mortality proportions between reference diagnoses and VA diagnoses in the validation sample. Additional descriptive analyses were conducted to understand the patterns of misclassification resulting in these biases in the performance of VA.

The observed biases in the performance of VA methods were used to adjust the findings from application of VA in the verification study. The numbers of deaths identified from each cause in the verification study were adjusted by misclassification patterns for the same cause as observed in the validation study as follows:

where *Verstudydeaths(j) *is the number of deaths identified with cause *j *in the verification study; and

*ValstudyMRprop (jk) *is the proportion of VA deaths from cause *j *in the validation sample that were classified to a reference diagnosis *k *based on medical records review.

***Adjusted deaths (k) ***is the estimate of deaths in the verification study sample due to cause *k*, and is the sum of the product of the above two terms across all deaths in the study sample for which reference diagnoses were available.

Finally, these adjusted numbers of deaths from each cause were used to derive estimates of cause-specific mortality for deaths outside hospitals in Thailand in 2005 by sex and age, aggregated across three broad age groups: 15-49 years, 50-74 years, and 75 years and over.

## Results

Out of the initial study sample of 11,984 deaths, VA methods were successfully applied in 9,817 cases (see Table One in [28]). VA interviews were completed for 6,328 out of 7,340 deaths in the study sample that had occurred outside hospitals, yielding the set of VA diagnoses for the verification study. Out of 4,644 deaths in the study sample that had occurred in hospitals, VA diagnoses were achieved for 3,489. However, corresponding medical record review diagnoses were only available in 2,558 cases, and these constituted the eventual matched sample for the VA validation study.

### Verification of cause for deaths outside hospitals

The observed response rate for deaths outside hospitals was 86% (6,328/7,340), approximately what was expected from the pilot study. Table 2 compares the age and sex distribution of deaths from the sampling frame (registration data for deaths outside hospitals) with the age-sex distribution of the field sample.

**Table 2:** Comparisons of age-sex distribution of deaths from registration data and the field sample of deaths outside hospital, Thailand, 2005

	Males		Females	
	
	% of deaths		% of deaths	
		
Age Group	Vital Registration	Field Sample	Vital Registration	Field Sample
<14 years	2.3	2.1	2.1	1.4
15-49 years	30.4	26.3	14.5	13.6
50-74 years	39.8	39.7	36.6	36
>75 years	27.5	31.9	46.8	49
All ages (100%)	143,021	3525*	111,822	2796*

*Excluding 2 male and 5 female deaths for which age could not be verified

Overall, the two sets of distributions are very similar. The slightly lower proportions of deaths in the field sample at younger ages are unlikely to significantly bias the study results because the total number of deaths at these ages in Thailand is comparatively small [37].

**Figure 1:**
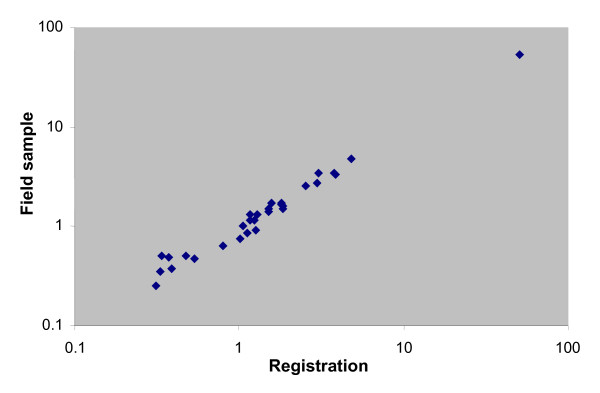
**Comparison of cause-specific mortality proportions from registration data with registered causes in the field sample, home deaths, Thailand, 2005**. Values on both axes plotted on a logarithmic scale. Causes include those listed in Table 5, plus septicemia, assault, hypertensive heart diseases, other infectious diseases, other digestive disorders, brain tumours, leukemia, breast cancer, cervical cancer, and colorectal cancer.

Similarly, the sampling procedure appears not to have introduced any substantial biases according to cause of death. Figure 1 shows the comparison between the proportionate mortality distributions of registered cause for the 30 leading causes of death from the sampling frame and from the field sample. The close concordance between the two distributions suggests that the 14% of cases lost to follow up were largely nondifferential by cause.

During data processing, the central team of experts noted that provincial coders had incorrectly coded the underlying cause in 22% of cases. However, in only 4% of cases did the resulting change in ICD code lead to an alteration of the ICD Mortality Tabulation List 1 category to which the death had been assigned (see Figure Two in [28] for details). This indicates that in general, the quality of certification and coding was of adequate quality.

The application of VA methods vastly improved the assignment of causes of death in the study sample compared with VR. In particular, the proportion of ill-defined causes was reduced from more than 53% to about 7%. At ages below 50 years, there were 238 deaths classified to ill-defined causes in the registration data for the field sample (185 males, 53 females). Among males, 13% were reassigned to HIV/AIDS by VA, and another estimated 7% to each of ischemic heart disease, stroke, other heart diseases, transport accidents, and alcohol abuse.

Only about 11% of adult male deaths aged 50 and over and 15% of female deaths in that age group that were originally classified as ill-defined remained so after the verification study (Tables 3 and 4). At ages 50-74 years, ill-defined deaths were reallocated by VA across a broad range of noncommunicable diseases in both males and females (Tables 3 and 4). Among the elderly (75 years and over), several such deaths were identified by VA to be due to communicable diseases, mainly tuberculosis, diarrheal diseases, and pneumonia, in addition to noncommunicable diseases. The inability of VA to ascertain specific causes for about 10% to 15% of deaths above age 50 may well have been due to the absence of clear symptom patterns for many deaths at these ages. Nonetheless, the vast reduction in diagnoses of no public health value following VA strongly suggests that the method could and should be applied routinely to all home deaths in Thailand.

**Table 3:** Proportionate reallocation (in%) by VA to specific causes for deaths coded to ill defined conditions (R00-R99) in the study sample for males, Thailand, 2005

Cause	50-74 yrs	75+ yrs	Total
Cerebrovascular diseases	7.1	9.2	16.3
Chronic lower respiratory diseases	3.4	7.4	10.8
Ischemic heart disease	4.5	3.2	7.7
Diabetes mellitus	2.3	2.3	4.6
Other genitourinary diseases	1.0	3.1	4.1
Other heart diseases	1.6	2.4	4.0
Liver cancer	1.7	2.1	3.8
Lung cancer	1.7	1.9	3.6
Tuberculosis	1.2	2.0	3.2
Hypertensive diseases	0.7	1.7	2.4
Pneumonia	0.6	1.7	2.3
Other digestive diseases	0.6	1.2	1.8
Diarrhoeal diseases	0.3	1.4	1.7
Liver diseases	1.0	0.3	1.4
Other specified causes	8.5	12.9	21.4
Ill defined conditions	2.3	8.5	10.9
Total (%)	38.6	61.4	100.0
**Total deaths**	**561**	**892**	**1453**

**Table 4:** Proportionate reallocation (in%) by VA to specific causes for deaths coded to ill defined conditions (R00-R99) in the study sample for females, Thailand, 2005

Cause	50-74 yrs	75+ yrs	Total
Cerebrovascular diseases	3.3	12.0	15.4
Ischemic heart diseases	2.5	5.0	7.5
Diabetes mellitus	3.2	4.1	7.3
Chronic lower respiratory diseases	1.7	3.0	4.6
Hypertensive diseases	1.3	3.0	4.3
Other heart diseases	0.5	3.5	4.0
Other genitourinary diseases	1.1	2.0	3.2
Pneumonia	0.3	2.7	3.0
Diarrhoeal diseases	0.5	2.3	2.8
Falls	0.4	1.7	2.2
Other digestive diseases	0.8	1.3	2.1
Liver cancer	0.8	1.1	2.0
Other cancers	1.3	0.7	2.0
Muscluloskeletal disorders	0.7	1.3	2.0
Other specified causes	6.5	15.3	21.8
Ill defined conditions	1.4	14.5	15.8
Total (%)	26	74	100.0
**Total deaths**	**439**	**1221**	**1660**

Along with the substantial reduction in the proportion of deaths originally assigned to ill-defined causes, the study also identified several important diagnostic differences among deaths assigned to specified causes in the vital registration data. Table 5 provides a summary of the extent of misclassification for the 20 leading causes of deaths outside hospitals in the study sample.

**Table 5:** Misclassification patterns suggested by verbal autopsy among the study sample of deaths outside hospitals, Thailand, 2005

		Verbal autopsy diagnoses (VA)				
				
Cause	As recorded in VR	Agreement with VR	Assigned to other cause	Assigned from other cause	Final total (VA)	Kappa
Ill defined conditions	3371	441	2930	32	473	0.11
Other cancers	307	45	262	75	120	0.19
Other genitourinary diseases	218	85	133	137	222	0.36
All other external causes	217	21	196	82	103	0.11
Liver cancer	213	178	35	237	415	0.55
Transport accidents	175	168	7	165	333	0.65
Diabetes mellitus	161	104	57	274	378	0.36
Other respiratory diseases	110	1	109	20	21	0.01
Diseases of the liver	108	29	79	84	113	0.24
Lung cancer	104	71	33	121	192	0.47
Cerebrovascular diseases	103	64	39	626	690	0.14
Other nervous system disorders	95	14	81	68	82	0.15
HIV/AIDS	94	86	8	172	258	0.48
Drowning	90	71	19	17	88	0.79
Suicide	83	67	16	49	116	0.67
Chronic lower respiratory diseases	82	53	29	310	363	0.22
Respiratory tuberculosis	72	28	44	108	136	0.26
Other heart diseases	72	13	59	162	175	0.09
Pneumonia	63	7	56	108	115	0.07
Ischemic heart disease	59	28	31	322	350	0.12
All other causes	531				1585	
**Total deaths**	**6328**				**6328**	

In addition to ill-defined conditions, a clear majority of deaths from several "other" categories were also reallocated to specific causes. The low *kappa *scores (< 0.4) for these, as well as some specific categories such as COPD, diabetes, and ischemic heart disease, indicate the low agreement between registration and VA data for these causes, taking into account the possibility of such agreement by chance. The observed *kappa *scores for some specific categories such as site-specific cancers and external causes at best suggest a moderate degree of agreement (0.4 to 0.7). To the extent that the VA diagnoses may be considered more accurate, given the detailed process for data collection, cause-of-death ascertainment, and selection and coding of underlying causes of death, these findings suggest poor reliability of causes of death in the registration data for nonhospital deaths from both specific and nonspecific causes.

Overall, the large numbers of deaths reclassified by VA from ill-defined causes and "other" categories result in an increase in the proportions of deaths ultimately classified to specific causes such as HIV/AIDS (from 1.4% to 4.4%), ischemic heart disease (IHD) (from 0.9% to 5.5% ), stroke (from 1.6% to 10.9% ), and transport accidents ( from 2.7% to 5.2%). These findings, however, need to be interpreted in the context of the performance characteristics of VA, as inferred from the *validation *study.

### Validation of VA methods

The reliability of findings from verification of registration data using VA methods is inherently dependent on the validity of the VA in the Thai context. The results from the validation sample of 2,558 deaths for which diagnoses from medical records review as well as VA were available, shown in Table 6, reveal several interesting findings. First, sensitivity scores were good (>75%) for some site-specific cancers and most external causes, indicating that if a death is diagnosed by VA to be from these causes, it is likely to be actually due to that cause. However, sensitivity was average (50%-75%) for major causes of death such HIV/AIDS, cerebrovascular and ischemic heart diseases, COPD, and diabetes.

**Table 6:** Validation characteristics of verbal autopsy procedures for 20 leading causes of hospital deaths in the study sample, Thailand, 2005

Cause of death	Medical record (MR) diagnoses	Verbal autopsy (VA) diagnoses	Validation scores for VA		
			
			Sensitivity	PPV	CSMF change in VA (%)*
Cerebrovascular diseases	269	285	69.1	65.3	6.5
Diabetes mellitus	158	224	63.9	45.1	41.8
Ischemic heart disease	203	199	49.8	50.8	-1.9
Transport accidents	185	199	97.8	91.0	7.6
Chronic lower respiratory diseases	143	147	61.5	59.9	2.9
HIV/AIDS	191	123	60.7	94.3	-35.6
Lung cancer	84	99	79.8	67.7	17.9
Diseases of the liver	112	93	44.6	53.8	-17.1
Malignant neoplasm of liver	65	88	78.5	58.0	35.3
Other genitourinary diseases	107	86	30.8	38.4	-18.8
Other heart diseases	64	65	15.6	15.4	0.0
Pneumonia	85	61	21.2	29.5	-28.2
Hypertensive diseases	58	54	12.1	13.0	-6.6
Respiratory tuberculosis	31	46	32.3	21.7	48.8
Colorectal cancer	33	45	84.8	62.2	36.4
Falls	35	39	60.0	53.8	11.7
Assault	33	38	90.9	78.9	15.5
Remainder of malignant neoplasms	37	34	27.0	29.4	-8.3
Other digestive disorders	53	34	20.8	32.4	-36.1
All other external causes	30	34	50.0	44.1	16.7
All other causes	582	565			
**Total deaths**	**2558**	**2558**			

*indicates the change in cause-specific mortality fractions (CSMF) from VA:

+ ve change indicates over diagnosis by VA;

- ve change indicates under diagnosis by VA

Sensitivity was poor (<50%) for deaths from the "other" categories, as might be expected, but was also poor for specific causes such as tuberculosis, hypertensive diseases, and pneumonia. While compensatory misclassification patterns within the validation sample tend to minimize the impact of these poor sensitivity scores on the net changes to cause-specific mortality proportions, as seen in the case of major causes such as ischemic heart disease, cerebrovascular disease, and COPD, this is not the case for other causes. Overall, VA tends to overdiagnose important causes such as diabetes, liver cancer, and tuberculosis, while undercounting deaths from HIV/AIDS, liver diseases, genitourinary (essential renal), and digestive system disorders, and, interestingly, pneumonia. These findings have important implications when using VA validation results to adjust findings from the verification study.

Table 7 shows the detailed misclassification patterns for important causes of death that result in these changes to cause-specific mortality proportions in the validation study sample. Almost all deaths classified to HIV/AIDS by VA were confirmed upon medical record review (116 out of 123 deaths), signifying a very high positive predictive value. However, another 75 deaths were classified to HIV/AIDS from other infectious conditions as well as noncommunicable diseases, indicating poor sensitivity as well as an undercount of HIV/AIDS by VA by about 35%. On the other hand, only 45% of deaths classified by VA to diabetes were confirmed (low PPV), with the others actually being cases of ischemic heart disease (13%), stroke (8%), renal failure (6%), and other conditions. Overall, the number of deaths classified to other conditions by VA that were actually found to be due to diabetes upon MR review were not as many as the VA misdiagnoses of diabetes, indicating that VA overcounts deaths from diabetes. Finally, although the difference in cause-specific mortality proportions from VA and MR diagnoses for ischemic heart disease is negligible, the matrix shows profound misclassification, with 50% of VA diagnoses of IHD being incorrect, and an equal number of deaths classified to other causes by VA actually being due to IHD upon medical record review.

**Table 7:** Discrepancies observed between verbal autopsy diagnoses and medical record review in the validation sample

Causes of death	Medical records diagnoses																
**Verbal autopsy diagnoses**	**5**	**20**	**25**	**52**	**66**	**67**	**68**	**69**	**74**	**76**	**77**	**79**	**80**	**81**	**84**	**All other causes**	**Total**

Tuberculosis (5)	10	9	2			1	1	1	3	6	4		2		3	4	**46**
HIV/AIDS (20)		116											2		1	4	**123**
Other infectious diseases (25)	1	8	8						2	1			1	1	1	6	**29**
Diabetes (52)		3	1	101	5	29	3	19	7	6	2	1	7	3	13	24	**224**
Hypertensive diseases (66)	1			7	7	10	1	5	5	1	1			2	9	5	**54**
Ischemic heart diseases (67)	2	4	1	14	11	101	13	6	4	7			4	2	7	23	**199**
Other heart diseases (68)				1	4	17	10	7	3	2	1		1		2	16	64
Cerebrovascular diseases (69)	2	1	1	13	3	15	10	184	9	5	1	1	3	1	4	36	**285**
Pneumonia (74)	2	5		2	4	1	3	2	18	6	1		1	2	3	11	**61**
COPD (76)	4	4	2		2	7	4	4	4	88	1	1	2	2	6	16	**147**
Other respiratory diseases (77)	1	2			1			1	2	2	3		1	1	2	1	**17**
Peptic ulcer (79)		1	1			3			1	1	1	10	5	5	2	2	**32**
Liver diseases (80)		7		1		2	2	3	3	1	1		50	7	2	14	**93**
Other digestive diseases (81)		1	1	1				3	1				2	11	2	10	**34**
Genitourinary diseases (84)		1		5	15	1	3	2	2			2	3	3	33	18	**86**
All other causes	8	29	8	13	6	16	14	32	21	17	5	1	28	13	17	836	**1064**
**Total**	**31**	**191**	**25**	**158**	**58**	**203**	**64**	**269**	**85**	**143**	**21**	**16**	**112**	**53**	**107**	**1022**	**2558**

These and other misclassification patterns observed in Table 7 provide evidence on the biases that result from the use of VA in cause-of-death ascertainment in the Thai context. Accordingly, the findings from the validation study were applied to adjust for overcount or undercount by VA in the verification study, under the assumption that these performance characteristics of VA derived from deaths in the hospital sample are applicable for deaths outside hospitals in Thailand.

Tables 8 and 9 show the final estimates of cause-specific mortality by age and sex for nonhospital deaths in Thailand after this further adjustment. Although deaths from ill-defined conditions are still the leading cause of nonhospital deaths at all ages in males and females, the magnitude of this category has been substantially reduced from the more than 50% observed in the registration data. Stroke, IHD, HIV/AIDS, and chronic lower respiratory diseases are leading causes of out-of-hospital deaths for both sexes, as are transport accidents for men and diabetes mellitus for women. These findings are similar to the mortality patterns derived for the sample of deaths that had occurred in hospitals [34] and reinforce the need for appropriate policy responses. This pattern would not have been at all evident from the raw, uncorrected registration diagnoses.

**Table 8:** Cause-specific mortality estimates (in%) by age for deaths outside hospitals in the study sample for males, Thailand, 2005

Cause of death	Age group				Total
		
	<15 yrs	15-49 yrs	50-74 yrs	>75 yrs	
Cerebrovascular diseases		0.7	4.5	3.6	8.9
Transport accidents	0.5	5.0	1.2	0.1	6.8
HIV/AIDS	0.2	5.7	0.6	<0.1	6.4
Chronic lower respiratory diseases		0.3	2.4	3.2	6.0
Ischemic heart disease		0.6	3.5	1.7	5.9
Liver cancer		0.8	3.1	0.9	4.8
Diseases of the liver		1.4	2.1	1.1	4.5
Lung cancer		0.3	2.2	1.0	3.4
Other genitourinary diseases		0.2	1.4	1.6	3.2
Diabetes		0.2	1.9	0.9	3.0
Suicide		1.8	0.8	0.1	2.8
Pneumonia		0.4	0.7	1.2	2.3
Drowning	0.6	0.8	0.4	0.1	2.0
All other external causes		0.9	0.7	0.4	1.9
Other heart diseases		0.4	0.7	0.8	1.9
Other malignant neoplasms		0.3	1.0	0.4	1.6
Assault		1.1	0.4	<0.1	1.5
Other digestive disorders		0.2	0.6	0.7	1.4
Hypertensive diseases		0.1	0.6	0.7	1.4
All other specified causes	0.7	3.8	8.3	5.8	18.5
Ill defined conditions		1.3	2.8	7.7	11.8
Total deaths (%)	2.1	26.3	39.7	31.9	100
**Total deaths**	**73**	**927**	**1398**	**1127**	**3523**

**Table 9:** Cause-specific mortality estimates (in%) by age for deaths outside hospitals in the study sample for females, Thailand, 2005

Cause of death	Age group				Total
		
	<15 yrs	15-49 yrs	50-74 yrs	>75 yrs	
Cerebrovascular diseases		0.4	3.3	7.3	11.0
Diabetes		0.4	4.2	2.6	7.2
Ischemic heart disease		0.3	2.5	3.8	6.5
HIV/AIDS	0.1	4.3	0.1	0.1	4.5
Other genitourinary diseases		0.4	2.3	1.7	4.4
Chronic lower respiratory diseases		0.1	1.6	1.9	3.8
Pneumonia		0.1	0.7	2.7	3.5
Liver cancer		0.5	2.2	0.7	3.3
Hypertensive diseases		0.1	1.4	1.6	3.1
Cervical cancer		0.8	1.8	0.3	2.9
Other heart diseases		0.1	0.5	1.8	2.4
Other digestive disorders		0.1	1.0	1.2	2.3
Diseases of the liver	0.1	0.3	1.0	0.7	2.1
Other malignant neoplasms		0.2	1.4	0.5	2.1
Lung cancer		0.2	1.3	0.5	2.0
Transport accidents	0.4	1.0	0.5	0.1	1.9
Musculoskeletal disorders		0.1	0.7	1.0	1.8
Falls		<0.1	0.5	1.2	1.7
All other external causes		0.4	0.4	0.9	1.7
All other specified causes	0.7	3.8	7.8	8.6	20.7
Ill defined conditions	0.1	0.1	1.1	10.0	11.2
Total deaths (%)	1.4	13.6	36.0	49.0	100
**Total deaths**	**38**	**380**	**1002**	**1376**	**2796**

## Discussion

Thailand is one of several countries that has a reasonably functional vital statistics system, yet the data it produces are of limited public health utility. This largely arises from the lack of medical opinion as to the registered cause of most deaths outside hospitals that constitute the majority of deaths in these countries. The reliance on lay reporting of the cause of death by relatives of the deceased leads to large numbers of such deaths being assigned to ill-defined or nonspecific causes, and contributes to uncertainty in the data on specific causes as well. In Thailand, local civil registrars are required to inquire about medical evidence on the cause of death from relatives at the time of death registration, usually discharge records from previous hospitalizations or notes from medical consultations during the illness preceding death. However, there is no systematic approach to such inquiry, and it is not adopted consistently across the country, adding to the uncertainty in registration data on causes of death.

Our study was designed to develop and test a detailed VA data collection instrument, including a comprehensive list of structured questions on symptoms and other relevant medical history, open text descriptions as told by the informant, as well as any relevant diagnostic information or treatment history available within the household. Our VA procedures also enabled us to ascertain the applicability of standard disease or condition-specific diagnostic guidelines that have been developed to help identify probable causes of death from VA responses [29,38]. Our data collection methods and the application of such diagnostic guidelines enabled ascertainment of the probable cause of death for the vast majority of deaths in the study sample. Feedback from physician reviewers of the VA responses suggested that the open narrative component of the questionnaire was essential in cause-of-death determination. Further research is required to determine whether local health personnel could be trained to apply these diagnostic guidelines for cause-of-death ascertainment, with a view toward routinely implementing such procedures to improve the quality of data on causes of death in registration data. There is some basis to conclude that they might. The provincial health authorities in Ubolrajthani (the province with the largest sample of deaths in this study) are already planning to implement the methods developed in this study to collect data on all deaths in the province in 2009 (Yawarat P., personal communication). The findings from their experiment will provide important evidence on the broader applicability and utility of these methods for improving cause-of-death diagnoses.

The most important limitation of our study lies in the underlying uncertainty of VA methods arising from recall and/or information bias in the responses to the VA interview, as well as the potential for inconsistency in the application of diagnostic guidelines by physician reviewers. Although we conducted a concurrent validation study, there could be selection bias in the hospital deaths included in the validation study because the characteristics of cases where the death occurred in a hospital may not be the same as for deaths from the same cause that occurred outside hospitals [39]. This selection bias could affect our assumption that the VA performance characteristics from the validation study are applicable in adjusting the findings from the verification study.

On the other hand, there could be certain cultural or language-specific issues that influence the comprehension of (and responses to) specific VA questions. These issues could be common to Thai society in general, and therefore influence VA responses for deaths in hospitals and homes in a similar manner, resulting in systematic biases common to all application of VA in Thailand. These issues need to be explored through in-depth sociological and anthropological research. In this study, we chose to test the utility of this additional adjustment using the validation study findings, and believe that this step yields more plausible cause-of-death estimates than without it. This is particularly apparent through the increase in the proportion of deaths from HIV/AIDS, which now approximates an independent estimate of deaths from HIV/AIDS in Thailand in 2005 developed by UNAIDS [40]. Also, the effect of gross systematic overcounting of diabetes by VA has been limited by this adjustment. For all other causes, these adjustments at best result in marginal differences to the overall estimates (see Figures Four and Five in [28]).

The development and application of VA methods specific to the Thai context are an important step toward the improvement of data quality from civil registration and vital statistics systems in Thailand. Further refinement of the questionnaire in terms of item reduction or modification would be useful, as would better methods and procedures to capture information from medical records for deaths that occur outside hospitals. This critical clinical information is likely to be available for a significant proportion of such deaths, given the better access to health care in Thailand, through universal health insurance schemes, as compared to other developing countries. Research in these areas is urgently required if there is to be an acceleration in the improvement of data quality on registered causes of death. Our study has added to the body of knowledge about the application of verbal autopsy methods in developing countries, but more importantly, has demonstrated the very significant potential of the method to reduce ignorance about the leading causes of death in populations where a large proportion of deaths occur outside hospitals.

## Competing interests

The authors declare that they have no competing interests.

## Authors' contributions

WP led the field work. CR designed the study along with ADL and YP. WP, CR, TA and JP contributed to data analysis. CR drafted the initial manuscript. All authors collaborated in developing the final manuscript and approved the final version.
